# Synthesis and antioxidant activity evaluations of melatonin based analogue indole-hydrazide/hydrazone derivatives

**DOI:** 10.1186/1753-6561-6-S4-P28

**Published:** 2012-07-09

**Authors:** AD Yılmaz, H Shirinzadeh, T Coban, S Suzen, S Ozden

**Affiliations:** 1 Department of Pharmaceutical Chemistry, Faculty of Pharmacy, Ankara University, Turkey; 2 Department of Pharmaceutical Toxicology, Faculty of Pharmacy, Ankara University, Turkey

## Introduction

Harmful effects of free radicals to the human body have been studied over the last decade. Overproduction of the free radicals can be responsible for tissue injuries that cause many health problems which include cancer, aging, heart diseases, neurological disorders, Alzheimer's disease, Huntington disease and so on. Melatonin (MLT), the main secretory product of the pineal gland is a well-known antioxidant and free radical scavenger. It is a neurohormone produced from the amino acid tryptophan. In our earlier studies, new MLT-based analogues with changes in the 5-methoxy and 2-acylaminoethyl groups of MLT were synthesized and tested for their in vitro antioxidant potency in the DPPH, superoxide dismutase and lipid peroxidation (LP) assays.

## Methods

In this study 5-chloroindole hydrazide/hydrazone derivatives were synthesized from 5-chloroindole-3-carboxaldehyde and phenyl hydrazine derivatives. All the compounds characterized and *in vitro* antioxidant activity was investigated against MLT and BHT.

## Results

The synthesized compounds were tested for their antioxidant activities using DPPH and superoxide radical scavenging and LP inhibitory activity tests. Ten of the synthesized compounds showed strong inhibitory effect on the superoxide radical scavenging assay. Almost all the tested compounds possessed strong scavenging activity against the DPPH radical scavenging activity with IC50 values (2 to 60 μM).

## Conclusions

MLT has redox properties because of the presence of an electron-rich aromatic ring system, which allows the indoleamine to easily function as an electron donor12,13,31. It is possible that making the indole ring more stable electronically helped to act as a better electro donor. MLT scavenges the radicals via nitrogen centred radical, the indolyl (or melatonyl) cation radical32. Introduction of an imine group in to the side chain increased the stability of the indole molecule by helping the delocalization of the electrons. This might help to have high free radical scavenging activity in the synthesized.

